# Isolated Cavernous Hemangioma: A Rare Benign Lesion of the Stomach

**DOI:** 10.4021/jocmr832w

**Published:** 2012-09-12

**Authors:** Murat Basbug, Ridvan Yavuz, Mahmut Dablan, Birol Baysal, Metehan Gencoglu, Yusuf Yagmur

**Affiliations:** a Department of Surgery, Diyarbakir Education and Research Hospital, 21400, Diyarbakir, Turkey; b Department of Gastroenterolgy, Diyarbakir Education and Research Hospital, 21400, Diyarbakir, Turkey; c Department of Pathology, Diyarbakir Education and Research Hospital, 21400, Diyarbakir, Turkey

**Keywords:** Hemangioma, Vascular disease, Gastric Hemangiomas

## Abstract

Gastric cavernous hemangioma is a relatively rare benign gastric disease. Here we report the case of a 25-year-old male patient who had been admitted complaining of epigastric pain and hematemesis. Preoperative imaging indicated that the mass lesion palpated in the epigastric region was a probable mesenchymal tumor of gastric origin. Due to the hypervascular nature and submucosal localization of the mass, we did not obtain definitive preoperative diagnosis by endoscopic biopsy. The histologic diagnosis of cavernous hemangioma was confirmed by post-resection histopathologial evaluation of the mass.

## Introduction

Intra-abdominal hemangiomas are rarely found outside of the liver [1]. Gastric haemangioma accounts for only 0.05% of all gastrointestinal (GI) neoplasms [2]. Isolated gastric hemangiomas generally manifest in routine clinical practice as epigastric pain and upper gastrointestinal bleeding of occasional recurrence. Endoscopic investigations hold a crucial position in the establishment of a diagnosis. However, with regard to the submucosal localization of gastric hemangiomas, their dense vascular nature and requirement for biopsy to confirm the diagnosis are among the challenges encountered. Endoscopic ultrasonography (EUS) and contrast-enhanced computed tomography (CT) play crucial roles in establishing a diagnosis. Total excision is the curative treatment for cavernous hemangioma. Here, we report the case of a 25-year-old male patient who was admitted due to hematemesis and epigastric pain.

## Case Report

**Figure 1:**
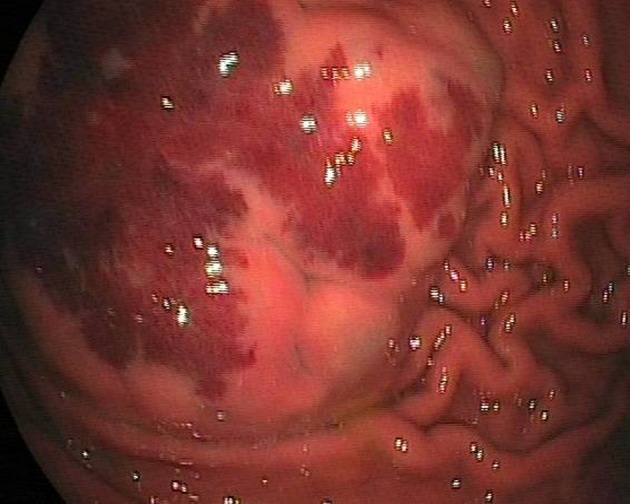
Endoscopic view of submucosal tumour of stomach showing lobulated submucosal tumor with dense vascularity.

**Figure 2:**
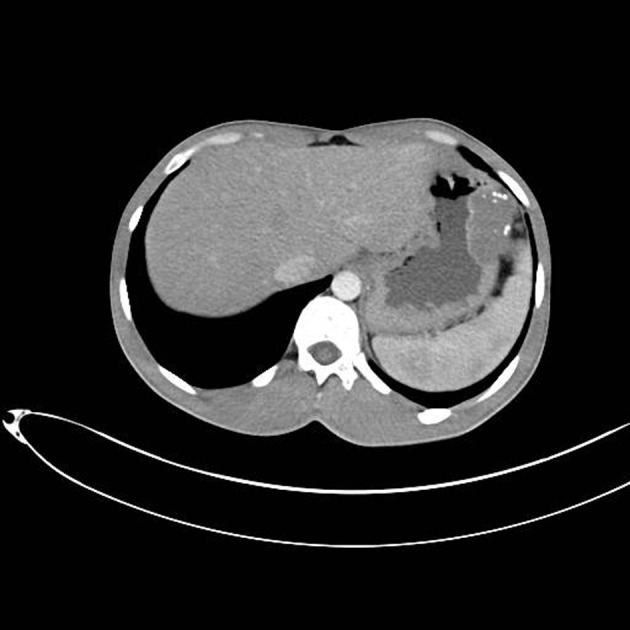
Computed Tomography image of gastric submucosal tumor. CT reveals an isodense and smooth contoured mass lesion that contained focal calcifications and was located on the greater gastric curvature in the left upper abdominal quadrant.

**Figure 3:**
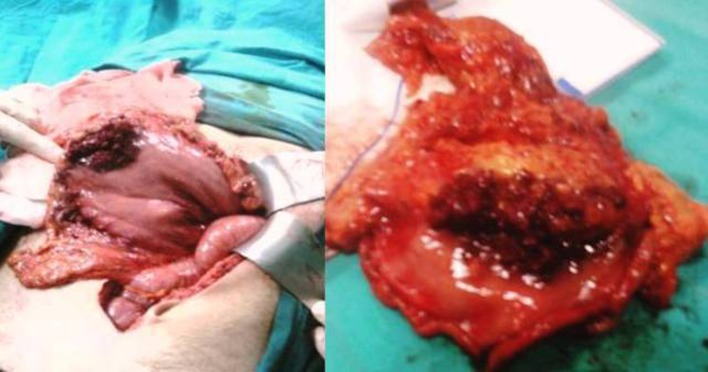
Intraoperative photograph of gastric tumour.

**Figure 4:**
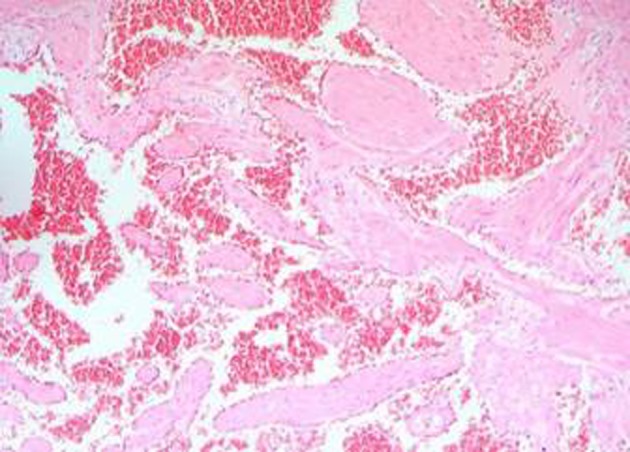
Histopathologic examination of the reseceted specimen. Numerous thick-walled blood vessels lined by endothelial cells and containing red blood cells. Appearance was characteristic of cavernous hemangioma (HE, × 100).

A 25-year-old male with a history of intermittent abdominal pain for the last six months was admitted to our emergency department complaining of intractable postprandial abdominal pain, along with bloody vomiting. Having been subject to a preliminary evaluation in our emergency department, the patient was transferred to the gastroenterology clinic for further investigation and treatment, where oral intake was stopped and replaced with parenteral replacement therapy. The patient’s medical background revealed nothing remarkable other than recurrent epigastric pain. He also had a history of receiving proton pump inhibitor drugs on an irregular basis. Upon physical examination, his general clinical status was good, with stable vital parameters. A palpable mass of 5 × 5 cm, along with tenderness in the epigastric region on palpation, was detected during abdominal examination. The laboratory test results, including liver and renal function tests, complete blood count, coagulation profiles and tumor markers were within normal limits. Upper GIS endoscopy revealed a submucosally located mass lesion of dense vascularity at the antrum-corpus junction on the greater gastric curvature. The appearance of the mass evoked the impression of a gastrointestinal stromal tumor of mesenchymal origin (Fig. 1). Accordingly, a biopsy was taken from this area. No definite focus of active bleeding was evident. In abdominal computed tomographic evaluation, an isodense and smooth contoured mass lesion of 52 × 25 × 32 mm that contained focal calcifications and was located on the greater gastric curvature in the left upper abdominal quadrant was detected (Fig. 2). Since the biopsy report indicated that the material was insufficient for a definitive diagnosis to be made, a deeper biopsy sampling was recommended. As such, Positron Emission Tomography (PET) was performed in an attempt to identify whether or not the mass was malignant. PET showed a smooth contoured mass of 5 × 5 cm, projecting from the gastric corpus into the lumen and containing scattered calcifications, which displayed no evidence of malignant transformation. A 6 × 4 × 5 cm mass was detected at the the greater gastric curvature on the anterior gastric surface during intraoperative exploration. The mass was not invading into adjacent tissues, and contained dense vascular structures (Fig. 3). No pathology associated with other intraabdominal organs was identified. Unluckily, no preoperative histopathological evaluation could be implemented due to the device for frozen sectioning in our hospital being out of order. The lesion appeared macroscopically to be benign. A wedge resection was first undertaken, since the lesion was consistent with a gastric hemangioma. The patient suffered no complications during the post-operative follow-up, and was discharged from the hospital on the seventh postoperative day. The pathology report was consistent with varicous dilatations of the submucosal veins and gastric cavernous hemangioma (Fig. 4).

## Discussion

Hemangiomas are congenital malformations that generally stem from the mesenchymal tissues. They may involve the skin, internal viscera, or both, with a predilection for the head and neck region (60%), the truncal region (25%), and the extremities (15%) [3]. The liver is the most frequent site of involvement among the internal viscera (0.4% - 20%) [4].

Hemangiomas of the gastrointestinal tract can be classified as capillary-, cavernous- or mixed-type, with a predilection towards the cavernous type in the gastrointestinal system. Gastric cavernous hemangiomas, on the other hand, are rare (0.05%). First defined by Lambers in 1893, gastric hemangiomas are most commonly encountered in adulthood, although they can occur in any age group [5]. Epigastric pain, dyspepsia and upper gastrointestinal system (GIS) bleeding are among the most frequent symptoms. Endoscopic assessment is of pivotal importance for preoperative diagnosis. Gastric hemangiomas provide a limited contribution to the final diagnosis due to their submucosal localization. Accordingly, USG, endoscopic USG, CT, abdominal magnetic resonance imaging (MRI) and angiography are important components of the imaging modality. Ultrasound reveals a solid mass with a heterogeneous multinodular appearance [6]. CT seems to be the most useful imaging method for establishment of a diagnosis, which typically shows phelobolitis and marked vascular enhancement with the ‘filling pattern’ characteristic of parenchymal haemangiomas [7]; moreover, it provides information regarding the extent and degree of invasion of the hemangioma [8]. MRI is also useful but is unlikely to contribute to the specific diagnosis. Angiography delineates the arterial supply of the tumor and is useful for confirming the diagnosis [9]. Angiography-directed embolization may also be beneficial in cases of acute bleeding. Endoscopic USG may prove helpful in characterizing the extent of vascularity of cavernous hemangiomas [10]. Positron Emission Tomography is eligible for benign/malignant differentiation of the mass in the preoperative period. The mass lesion and all abdominal cavities were evaluated by abdominal CT in our patient, revealing no additional intraabdominal pathology other than the aforementioned mass lesion in the upper abdominal region and bilateral millimetric nephrolithiasis.

The role of endoscopic biopsy in the preoperative histopathological diagnosis of hemangiomas is limited owing to the submucosal localization and dense vascular nature of such lesions [11]. As such, we were unable to establish a definitive diagnosis by means of endoscopic biopsy.

Other gastric submucosal masses, especially those of gastrointestinal stromal tumors, leiomyomas, lipomas, varicous vessels and carcinomas should be kept in mind in the differential diagnosis of cavernous hemangiomas due to their frequent submucosal localization. Biopsy may yield equivocal results for especially small lesions covered by normal mucosa. Assessment of such lesions by endoscopic USG becomes increasingly significant. Boyce et al. evaluated 91 patients with gastric submucosal tumors by EUS, reporting that endoscopic ultrasound was useful for the evaluation of upper gastrointestinal submucosal mass lesions [12]. Due to the lack of such a facility in our hospital, the submucosal lesion in our patient could not be evaluated. As for larger lesions, probable mucosal ulcerations and polypoid formations are likely to facilitate the establishment of a definitive diagnosis by endoscopic biopsy. Cavernous hemangiomas should be considered upon observation of dense vascular varicous dilatations in the submucosal area.

Surgery is the curative treatment. Accordingly, wedge resection, partial or total gastrectomy constitute the standard treatment modalities for isolated gastric hemangiomas [13]. A surgeon should meticulously evaluate the lesion and determine the appropriate procedure according to lesion localization. For this reason, we performed a wedge resection in our patient owing to the lesion being located on the greater curvature and its smooth contours.

Endoscopic resection may be preferred for submucosal lesions less than 2 cm in diameter, which can easily be removed from the muscularis propria [14]. Arafa et al. reported a case from whom they extracted a hemangioma around 14 mm in diameter, which was located on the posterior antral wall [15]. In parallel with the advent of new endoscopic instruments and techniques, endoscopic resection is becoming increasingly widespread.

In conclusion, gastric hemangiomas are rare and benign tumors of the stomach. Use of endoscopy in conjunction with CT is beneficial for diagnosis. The role of endoscopic biopsy, however, remains limited. Endoscopic resection can be undertaken in selected cases. Surgery is the curative treatment. Moreover, surgical treatment along with histopathological studies occupy an important place in the establishment of a definitive treatment.
